# Supplementary material for: Distributed forward Brillouin sensor based on local light phase recovery
Source: Nat Commun. 2018 Jul 31;9:2990. doi: 10.1038/s41467-018-05410-2 (PMC6068146; doi:10.1038/s41467-018-05410-2)
Supplement: Supplementary file 1 — Supplementary Information [file 41467_2018_5410_MOESM1_ESM.pdf]

Distributed forward Brillouin sensor based on local light phase recovery

Desmond M. Chow, Zhisheng Yang, Marcelo A. Soto†, Luc Thévenaz

EPFL Swiss Federal Institute of Technology, Institute of Electrical Engineering, SCI-STI-LT Station 11, CH-1015 Lausanne, Switzerland.

†*Permanent address: Department of Electronic Engineering, Universidad Técnica Federico Santa María, 2390123 Valparaíso, Chile*

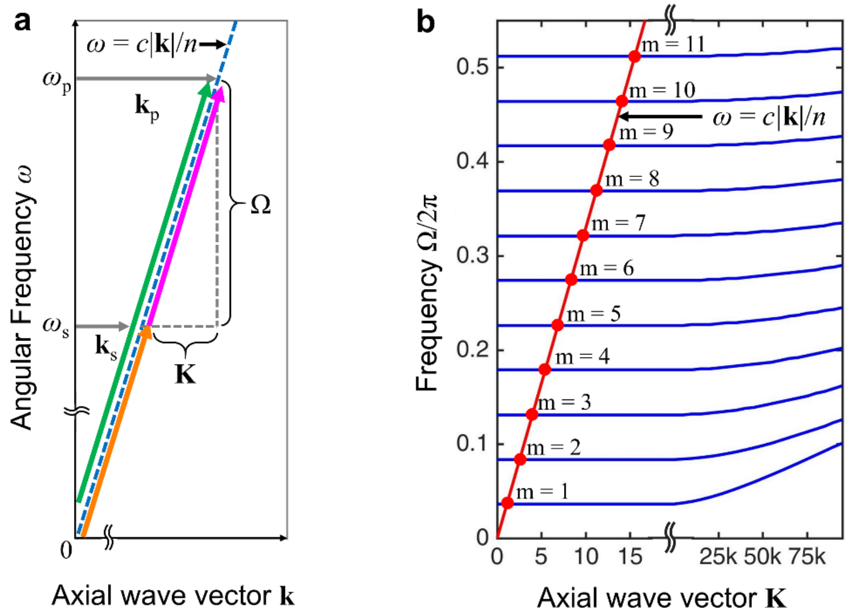

Supplementary Figure 1| Simulated guiding properties of the transverse acoustic waves in standard single-mode fibre. The dispersion diagrams describe: (a) The phase matching between the activating lights (green and orange arrows) and the transverse acoustic wave (magenta arrow) through forward stimulated Brillouin scattering (FSBS). (b) The transverse acoustic wave is activated when the frequency difference of the activating lightwaves $\Delta\omega = \omega_p - \omega_s$ matches the resonant frequency Ω of one of the transverse acoustic modes (blue lines) as indicated by the red dots. Mathematical symbols are defined as follows: ω_p, ω_s, and Ω are the frequencies of the pump, probe lightwaves and the transverse acoustic wave, respectively; $\mathbf{k}_p$, $\mathbf{k}_s$, and $\mathbf{K}$ are the axial wave vectors of the pump, probe lightwaves and the transverse acoustic wave, respectively; n is the effective refractive index; m is the radial mode number.

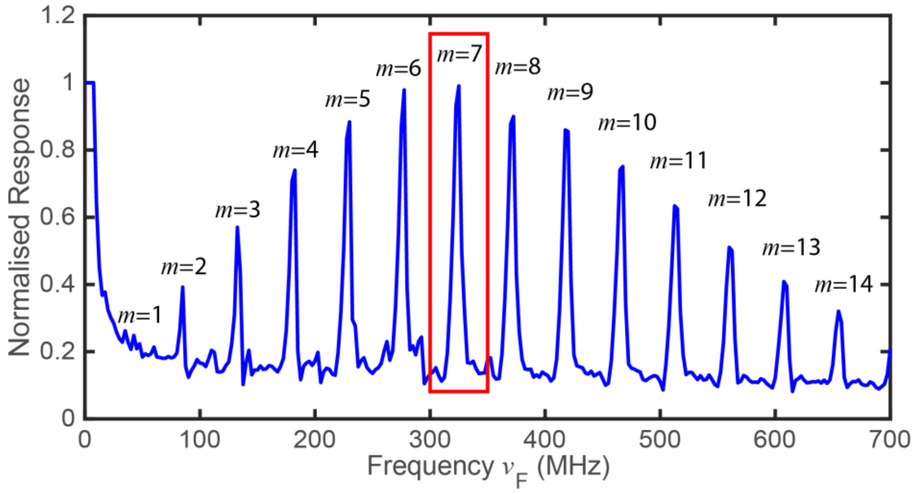

Supplementary Figure 2| Spectrum of forward stimulated Brillouin scattering in standard single-mode fibre. The integrated spectrum of forward stimulated Brillouin scattering (FSBS) in a 30 m uncoated standard single-mode fibre that is obtained through the Sagnac fibre inteferometry detection method with frequency sweeping[3]. The FSBS spectrum shows radial transverse acoustic modes with varying excitation efficiencies. The spectral profile of the most efficient FSBS resonant mode (7th mode, in red box) is measured in the proposed distributed FSBS analysis. The FSBS spectrum is normalised by the peak power of the most efficient FSBS mode.

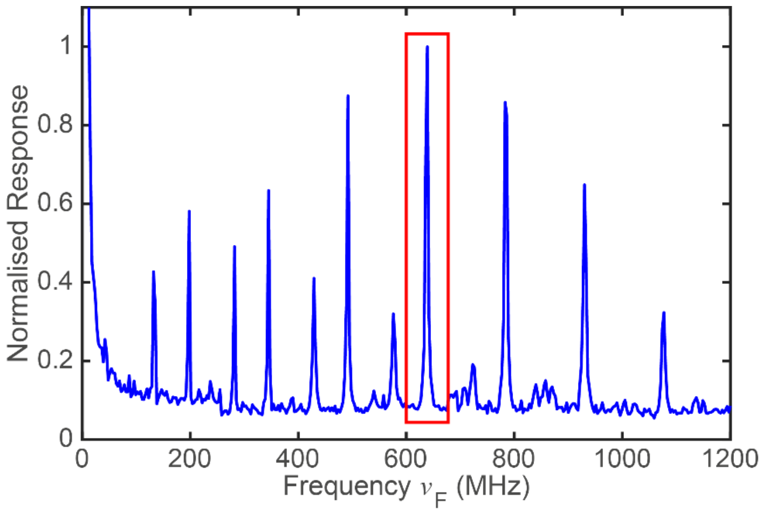

Supplementary Figure 3| Spectrum of forward stimulated Brillouin scattering in 8 µm-thickness polyimide coated fibre. The integrated spectrum of forward stimulated Brillouin scattering (FSBS) for an 80 µm-diameter single-mode fibre coated with 8 µm-thickness polyimide layer. The most efficient FSBS resonance (indicated in red box) is selected for the demonstration of acoustic impedance sensing. The FSBS spectrum is normalised by the peak power of the most efficient FSBS mode.

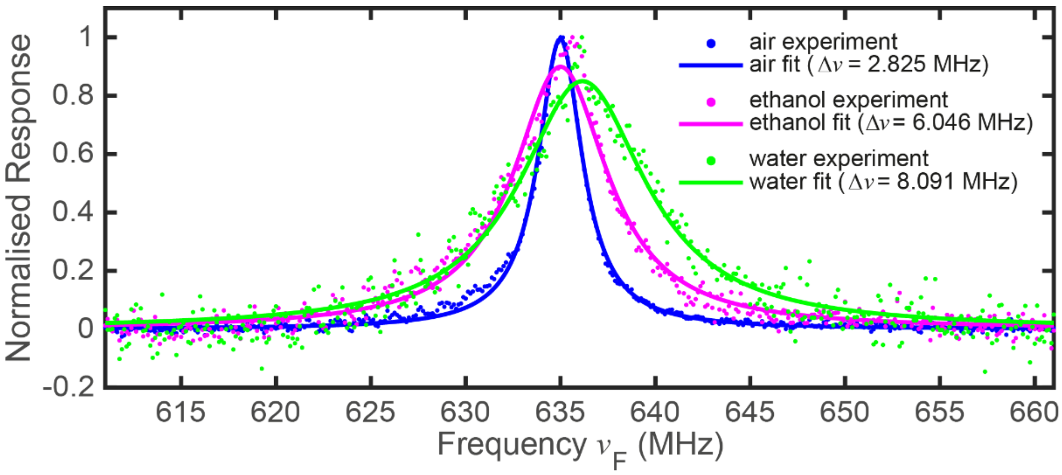

Supplementary Figure 4| Effect of different external media on the resonant linewidth of forward stimulated Brillouin scattering. The measured spectra of forward stimulated Brillouin scattering (FSBS) around the selected resonant mode. The resonant linewidth broadens when the entire fibre together with its protective coating is fully immersed in ethanol and water. The measured spectra are fitted with a Lorentzian function to retrieve their respective resonant linewidths.

Supplementary Table 1| Calculated and measured resonant frequencies of the radial acoustic modes in standard single-mode fibre.

Mode *m*	Calculation (MHz)	Measurement (MHz)
1	36.5	35.0
2	83.79	85.0
3	131.21	132.5
4	178.79	182.5
5	226.36	230.0
6	273.94	277.5
7	321.51	325.0
8	369.09	372.5
9	416.67	420.0
10	464.24	467.5

Supplementary Note 1

Guiding properties of the radial transverse acoustic modes in standard single-mode fibre

A standard single mode fibre (SMF) has cylindrical structure that confines the acoustic waves into discrete longitudinal, radial, torsional and flexural vibrational modes that can be stimulated via electrostriction. The induced acoustic modes present in the forward light scattering are the radial modes R_{0m} and the mixed torsional-radial modes TR_{2m}. The characteristic solutions of the radial modes y_m could be obtained analytically from the boundary condition of the fibre structure[1]:

$$(1-\alpha^2)J_0(y)-\alpha^2 J_2(y)=0 \qquad \text{(Supplementary Equation 1)}$$

where α is the ratio of the shear acoustic velocity V_s to the longitudinal acoustic velocity V_d, $\alpha = V_s / V_d$ (V_s = 3740 ms^{-1} and V_d = 5950 ms^{-1} for fused silica). The solutions are based on the Bessel functions J_n due to the axisymmetric shape of fibre. The eigenfrequencies f_m of the R_{0m} modes[1] are given by

$$f_m = V_d y_m / (2\pi a) \qquad \text{(Supplementary Equation 2)}$$

where a is the optical fibre radius. The calculated resonance frequencies of R_{0m} modes in the standard SMF (a = 62.5 µm) are listed in Supplementary Table 1 alongside with the experimentally measured values. The small experimental deviations are within the tolerance range of the fibre diameter.

The guiding characteristics of the transverse acoustic waves in a standard optical fibre is described here with the dispersion diagram of the participating optical and acoustic waves (Supplementary Figure 1) that is numerically simulated using the elastodynamic equation[2]. For the forward stimulated Brillouin scattering (FSBS) process, two co-propagating optical waves with frequencies ω_1 and ω_2, respectively, couple to the transverse acoustic modes that satisfy the phase matching conditions, $\Omega_m = \Delta\omega = \omega_1 - \omega_2$ and $\mathbf{K}(\Omega_m) = \Delta\mathbf{k} = \mathbf{k}(\omega_1) - \mathbf{k}(\omega_2)$ (see Supplementary Figure 1 (a)). For small axial wave vector $\mathbf{K}$, the phase velocities of the transverse acoustic waves are extremely high whereas their group velocities are negligibly small (blue lines in Supplementary Figure 2 (b)). Thus, the phase matching condition is relaxed because a broad range of acoustic phase velocities $V_a = \Omega/|\mathbf{K}|$ can match with the group velocity of the guided light $v_g = \Delta\omega/\Delta|\mathbf{k}|$ for a particular acoustic mode. Since the group velocity of light is close to its phase velocity for standard SMF in telecom wavelengths, the transverse acoustic waves propagate along the fibre at the same axial velocity as the guided light.

The FSBS spectrum can be observed experimentally using a non-distributed technique with optically isolated pump and probe waves[1]. The pump is an intense light that stimulates the transverse acoustic waves whereas the probe is configured as an interferometer to convert phase shifts induced by the transverse acoustic waves into intensity changes measurable by a photodetector. A specific frequency sweeping method[3] is used here to measure the integrated FSBS spectrum of standard SMF, the result is shown in

Supplementary Figure 2. Due to the partial overlapping between the optical and acoustic fields, the peak intensities of the transverse acoustic modes vary across the spectrum. The most efficient FSBS resonant mode is the 7th mode which is indicated in the red box in Supplementary Figure 2. This mode is selected and its distributed spectral profile along the fibre is measured and analysed.

Supplementary Note 2

Acoustic impedance sensing using optical fibre with a thin polymer coating

Stripping the sensing fibre of its coating is a simplified case for demonstrating the concept of distributed FSBS spectrum measurement technique. The standard SMF has acrylate coating with thickness of ~62.5 µm, which attenuates the transverse acoustic wave significantly before the reflection at the coating-surroundings boundary. However, a fibre with a thin coating layer allows the transverse acoustic waves to penetrate with insignificant loss, and at the same time, maintains the fibre's mechanical strength. Here, a commercial 80 µm-diameter single-mode fibre coated with 8-µm polyimide layer is used to measure the acoustic impedance of a material surrounding the fibre. The transverse acoustic spectrum is obtained by using Sagnac interferometry interrogation technique[3,4] and the full measured spectrum is shown in Supplementary Figure 3. A complex FSBS spectrum can be observed due to the double boundary structure of the fibre, however sharp distinct resonance peaks keep well defined. A resonance peak with the highest efficiency (ν_{res} = 635 MHz) is selected for acoustic impedance sensing. The fibre bulk, together with its thin polyimide coating, are immersed in different liquids. The resulting FSBS resonant spectra of the selected mode in air, ethanol and water are shown in Supplementary Figure 4. The acoustic impedances of the surrounding liquids can be retrieved from the resonant linewidth broadenings by using the acoustic analogy of optical thin film reflection.

Supplementary References

1. Shelby, R., Levenson, M. & Bayer, P. Guided acoustic-wave Brillouin scattering. *Phys. Rev. B* **31,** 5244 (1985).
2. Beugnot, J.-C. & Laude, V. Electrostriction and guidance of acoustic phonons in optical fibers. *Phys. Rev. B* **86,** 224304 (2012).
3. Chow, D. M., Soto, M. A. & Thévenaz, L. Frequency-domain technique to measure the inertial response of forward stimulated Brillouin scattering for acoustic impedance sensing. in Optical Fiber Sensors Conference (OFS), 2017 25th 1–4 (IEEE, 2017).
4. Antman, Y., Clain, A., London, Y. & Zadok, A. Optomechanical sensing of liquids outside standard fibers using forward stimulated Brillouin scattering. *Optica* **3,** 510–516 (2016).
